# The Relativity of Indeterminacy

**DOI:** 10.3390/e23101326

**Published:** 2021-10-11

**Authors:** Flavio Del Santo, Nicolas Gisin

**Affiliations:** 1 Institute for Quantum Optics and Quantum Information (IQOQI-Vienna), Faculty of Physics, University of Vienna, A-1090 Vienna, Austria; 2 Department of Applied Physics, University of Geneva, 1211 Geneva, Switzerland; nicolas.gisin@unige.ch; 3 Schaffhausen Institute of Technology (SIT), 1211 Geneva, Switzerland

**Keywords:** fundamental indeterminism, special relativity, finite information

## Abstract

A long-standing tradition, largely present in both the physical and the philosophical literature, regards the advent of (special) relativity—with its block-universe picture—as the failure of any indeterministic program in physics. On the contrary, in this paper, we note that upholding reasonable principles of finiteness of information hints at a picture of the physical world that should be both relativistic and indeterministic. We thus rebut the block-universe picture by assuming that fundamental indeterminacy itself should also be regarded as a relative property when considered in a relativistic scenario. We discuss the consequence that this view may have when correlated randomness is introduced, both in the classical case and in the quantum one.

## 1. Introduction

In recent years, new attention has been brought to the problem of having infinite quantities in physical theories [1,2], with a special emphasis on the connection between physics and information [3,4,5,6,7,8,9,10,11]. (Many of these approaches stem from, inter alia, Landauer’s pivotal work, which supports the view that “information is physical” [12]). In particular, in previous studies [4,5,8,9,11,13], we pointed out that the usual understanding of classical (nonrelativistic) mechanics rests on the unwarranted implicit assumption of “infinite precision”, namely, that every physical quantity has an actual value with its infinite determined digits (which formally translates into the assumption that physical variables take values in the real numbers). This led to the general, supposedly uncontroversial, understanding of classical physics as a fundamentally deterministic theory.

To overcome this problem of “infinities”, in Refs. [4,5], we have assumed the “finiteness of information density” (i.e., finite regions of space can only contain finite information) as a foundational principle, and provided a model in which physical variables do not take values in the real numbers, but rather, in what we have called “finite information quantities” (FIQs). These are mathematical entities whose digits are not infinitely determined at every instant of time, but only the objective probability (propensity) of each digit to take a certain value is determined (and evolves in time). We then showed how the assumption of FIQs together with the existence of chaotic systems leads to an alternative fundamentally indeterministic interpretation of classical physics.

This led us to assume a primitive notion of “creative” time that openly contrast a widely accepted view according to which time is an evolution parameter in a set of deterministic laws of physics. The latter view about time has particularly crystallized among physicists with the advent of special relativity, which proposes a geometrization of space-time in a single “block-universe picture”. This fostered the conviction that time is a coordinate to describe events that are bound to be fully predetermined in the block-universe, and leaves no space for genuine indeterminism in physics.

In this paper, we show that upholding a principle of finiteness of information hints at a picture of the physical world that should be both relativistic and indeterministic. We thus propose a new way out of the conundrum of reconciling indeterminism and the flow of time with (special) relativity—both in the classical block-universe picture and in its quantum adaptation (Page–Wootters [14] and Many-World views [15]). It is the aim of this paper to show that today’s physics, and in particular, the theory of relativity, does not lead to accepting the block-universe as a necessity, but there exists at least an alternative consistent way to fit fundamental indeterminism within relativity. In particular, we rephrase by means of a physically intuitive scenario a now classical argument independently put forward by Rietdijk [16] and Putnam [17] that “illustrate(s) the power of the block-universe picture” [18] by allegedly showing the incompatibility between (special) relativity and indeterminism. We then discuss its implicit assumptions, pointing out that one of them—which seems innocuous in Newtonian physics—is problematic in special relativity, thus making their argument untenable. As we shall see, the consequences of putting together the concepts of indeterminism and relativity would lead us to conclude that the state of determinacy of a physical (random) variable is relative as well. The relativity of indeterminacy will be analyzed in the different scenarios of classical independent randomness, classically correlated randomness, and quantum correlated randomness.

## 2. Relativity from Information Principles

The theory of special relativity can be regarded as stemming from an attempt to remove a problem with infinities that is intrinsic in classical physics. In fact, in Newtonian mechanics, the interaction of particles is described by the potential energy of interaction, which is a function of only the positions of the interacting particles; hence, as stressed also by Landau and Lifshitz, such a theory “contains the assumption of instantaneous propagation of interactions” [19]. Thus, they motivated the development of the theory of special relativity (SR) so as to overcome a long-standing problem of “infinities” (the infinite speed of propagation of the interactions in this case), which affected classical physics. (Note that SR only deals with mechanics and electromagnetism, whereas to address similar problems about gravity, one has to consider general relativity).

Remarkably, the problem of infinities also raised by Landau and Lifshitz can be addressed by invoking the same principle of “finiteness of information density” introduced in [5], as recalled in the introduction, thus assuming it as an alternative axiom from which SR can be derived. Consider the following two axioms:

**P1**— **Principle of relativity**: The laws of physics have the same form in every inertial frame of reference.
**P2**— **Principle of finiteness of information density**: A finite volume of space can only contain a finite amount of information. (Note that assumption P2 is supported, in the context of general relativity, by *Bekenstein’s bound* [20], which intuitively states that since information is associated with a certain amount of energy, unbound densities of energy would degenerate into black holes).

From P2, one infers that only a finite amount of information can be transmitted in a finite time; otherwise, this would require to “move” an infinite volume of space. Hence, the *signal velocity* (i.e., the speed of propagation of information) necessarily needs to be finite too. Now, if there is a maximal velocity, from P1, this must be the same in every inertial reference frame [19]. (Note that, from P2, it is only possible to infer that the signal velocity cannot be infinite, but this does not mean that there is a single maximal value thereof. In fact, in principle, it is possible that there is a set of different signal velocities, each of them finite if P2 holds, but without a maximum (i.e., the set is unbounded). Despite this theoretical possibility, we restrict our analysis to the case where there exists a maximal velocity). The former considerations are enough to derive the whole theory of special relativity (possibly with the further but quite innocuous assumptions of the homogeneity of space and time and isotropy of space). Incidentally, note that the fact that this maximal signal velocity is identified with the speed of light in a vacuum does not follow from physical principles, nor is it required at all to derive SR. (However, a body moving at the maximal signal velocity would be required by SR to be massless (see e.g., [21], p. 589). This, together with the empirical demonstration that Maxwell’s equations are invariant under Lorentz transformations, leads to the identification of the maximal velocity with the speed of light in vacuum, *c*. Note that *c* is not only identified with the speed of light but it also appears as a natural constant in several other theoretical frameworks (see [22]), most notably, as the speed of propagation of gravitational waves in vacuum. The numerical equivalence of these speeds has been recently empirically verified with high accuracy [23]).

The Principle of finiteness of information density (P2) has, thus, two physical consequences: On the one hand, it imposes that, in general, the values taken by physical quantities at future times (such as the position of a particle) are not fully determined by their present state; consequently, physical variables do not have determined values at all possible times (or, alternatively, that the truth value of certain empirical statements, such as “a particle is located at $\bar{x}$ at a certain instant $\bar{t}$”, is indeterminate). On the other hand, P2 also imposes a dynamical limit to the propagation of information (signal velocity), which, together with P1, allows us to fully derive the theory of SR. Hence, upholding the Principle of finiteness of information density gives us a hint that physics should be at the same time indeterministic and relativistic. However, are these two worldviews compatible?

In what follows, we will show that special relativity and fundamental indeterminism can be reconciled by postulating the existence of true random events—i.e., genuine acts of creations whose outcome is indeterminate before it actually happens—whose indeterminacy is to be considered a relative property as well.

## 3. Indeterminacy is Relative

### 3.1. Locally and Independently Generated Randomness

To discuss indeterminism in the framework of SR, and rephrase Rietdijk’s and Putnam’s argument, we introduce the concept of a True Random Number Generator (TRNG), by which we mean an abstract device that outputs genuinely random bits. Namely, before each bit is output, its value is not only unknown (epistemic uncertainty), but it actually has no determined value (ontic indeterminacy), even though there might be a probability distribution associated with the outcome to be realized (in principle, both at the epistemic and at the ontic level). More precisely, even having complete knowledge of (i) the *state*—i.e., the values of all the variables that may influence the outcome of the TRNG (this can be, in principle, everything that lies in the past light-cone of the event associated to the generation of the bit)—and (ii) of the *dynamical laws* that rule the evolution of each and every of the said variables, there is no way to predict with certainty which will be value of the bit output by the TRNG, not even in principle (see Table 1).

This is what we mean by true randomness, which clearly entails indeterminism. In what follows, we will be concerned solely with the (ontic) indeterminacy that each bit has before it is output and acquires a definite value (on the contrary, we will not consider what dynamical laws—deterministic or otherwise—govern the evolution of the considered systems). This very concept can be expressed in terms of truth values of empirical propositions, such that the statement, e.g., “the value of the bit *j* output by the TRNG at time $\bar{t}$ (in a certain reference frame) is j=0” has a definite truth value, either true or false, only after $\bar{t}$, whereas it was (ontologically) indeterminate before, i.e., its truth value is neither true nor false (see also [24]). (We refer to propositions (or statements) as *empirical* because they are about the values taken by physical variables, i.e., outcomes of hypothetical experiments; however, in principle, we will not immediately disregard those statements that refer to indirectly empirically accessible quantities (e.g., a joint proposition about two outcomes of experiments conducted in spacelike separation), which instead would be meaningless to genuine empiricists because they are unverifiable). Note that this makes the law of the excluded middle of classical logic fail, so it relates the concept of indeterminacy to mathematical intuitionism (see [11,25]).

**Figure 1:**
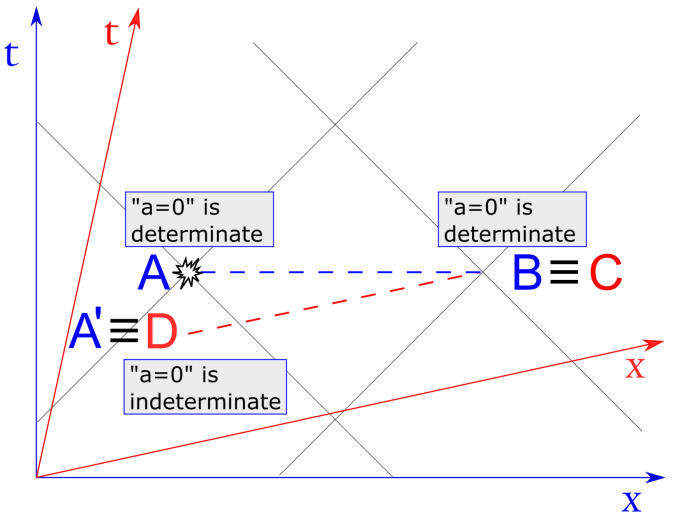
Space-time diagram (in 1 + 1 dimensions) illustrating Rietdijk’s and Putnam’s argument for the alleged incompatibility between special relativity and indeterminism. The observers Alice (A) and Bob (B) are at rest in the blue reference frame, whereas Charlie (C) and Debbie (D), also at relative rest, move at a constant speed in the positive *x* direction (their transformed reference frame is depicted in red). The blue dotted line represents the plane of simultaneity in Alice’s and Bob’s rest frame at the instant in which Alice’s True Random Number Generator outputs the bit *a* (i.e., when it becomes determinate; event A). In the moving reference frame (red), however, when Charlie overlaps with Bob, he is simultaneous with Debbie, which in turn overlaps with Alice’s past (event A’) when *a* was not yet determinate.

Turning now to SR, with reference to Figure 1, let us consider four inertial observers—Alice, Bob, Charlie, and Debbie. Assume that Alice has a TRNG that outputs a fresh random bit every minute (locally in her inertial reference frame). Assume also that Bob, at rest in Alice’s frame, is located at a certain fixed distance from Alice, e.g., one minute away at light speed (one light-minute). Charlie and Debbie, at relative rest, move at a constant speed *v* with respect to Alice’s and Bob’s frame. All parties know that Alice’s TRNG outputs a fresh random bit every minute in her frame, i.e., every $1/\gamma =1/\sqrt{1-v^{2}/c^{2}}$ minutes in Charlie’s and Debbie’s reference frame. Moreover, the positions and velocity *v* are tailored such that at 1:00 p.m. in both reference frames, Charlie’s position lies on Bob’s world-line (point B≡C in Figure 1), while Debbie’s position overlaps with Alice’s world-line (point $A^{\prime }\equiv D$ in Figure 1).

Let us denote by a∈{0,1} the bit output by Alice’s TRNG at 1:00 p.m. in her reference frame. As explained above, by the very nature of a TRNG, at any time instant before 1:00 p.m., the proposition “a=0” has no truth value for Alice (nor for any possible observer for that matter) but rather, is fundamentally indeterminate. Yet, at 1:00 p.m., Alice’s TRNG outputs a fresh bit that now becomes determined for Alice. Is the value of this bit determined for Bob as well? One may be tempted to answer in the positive (as in fact, Rietdijk and Putnam do), since the events happening at Alice’s and Bob’s locations, respectively, are simultaneous in their rest frames. Of course, Bob may not know the value of *a* because Alice may not have communicated it, or, more fundamentally, because Bob is spacelike separated from Alice; however, does the proposition “a=0” have a truth value or is it indeterminate for Bob?

Since at 1:00 p.m., Bob and Charlie are next to each other—ideally they exactly overlap at the same location—it is plausible to assume that the proposition “a=0” has the same truth value for both of them (since this is empirically testable, there is actually no room for any alternatives at the ontological level). Hence, let us assume it has a determinate truth value for Charlie as well. However, by applying the same reasoning as before, this implies that the proposition also has a truth value at all locations simultaneous to Charlie in his inertial frame, thus, it must be determinate for Debbie too. Yet, given the relative motion between the two reference frames, Debbie overlaps with Alice’s world line at the space-time point $A^{\prime }$, where her TRNG has not yet outputted the bit. Hence, following this chain of inferences, “a=0” had already a definite truth value before Alice’s TRNG outputs the bit *a*, which is a contradiction with the assumption of a TRNG. From this contradiction, Rietdijk and Putnam concluded that, in general, the structure of SR does not allow for indeterminate events.

The previous argument, however, relies on two assumptions that seem prima facie innocuous, which originate from an intuitive extension of classical concepts to a relativistic scenario. In fact, these assumptions are introduced to characterize what it means for two (or more) observers to share a determinate reality (in terms of truth values of propositions). Their assumptions can be made explicit as follows:

1. *Local reality*: Any two observers that locally overlap attribute the same truth values to empirical propositions (including the value “indeterminate”).
2. *Present reality*: Any two distant observers at relative rest attribute the same truth values (including the value “indeterminate”) to empirical propositions about present events (i.e., lying on the same plane of simultaneity in their rest frame).

However, while the former of these assumptions may still be upheld in SR because any two observers can operationally verify the consistency of their attributed truth values locally, the latter assumption becomes questionable. Indeed, we maintain that the finiteness of the maximum speed of propagation of any piece of information renders also determinacy (i.e., the definiteness of the truth values of empirical statements), a property relative to specific regions of space-time. This can be seen by taking any binary function (e.g., the sum modulo 2) of the statements describing the outcomes of local physical processes taking place at distant locations (e.g., the output bits of two distant TRNG’s). Thus, it becomes a straightforward assumption that determinacy itself propagates in space at the maximal signal velocity, i.e., instantaneously in Newtonian physics, but at a finite speed (that of light) according to relativity. This is where Rietdijk and Putnam’s argument fails, in the assumption that truth values are always shared by observers lying on the same plane of simultaneity, even though this is not verifiable except for the region enclosed within the intersection of their future light-cones. Hence, in short, (in)determinacy is relative.

**Figure 2:**
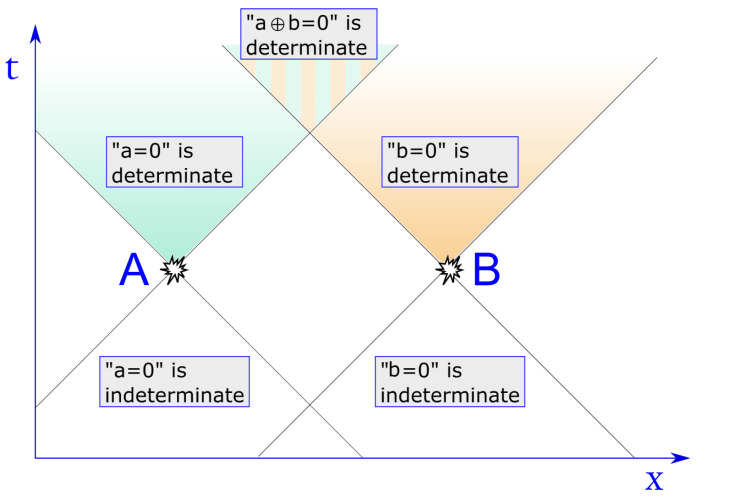
Space-time diagram (in 1 + 1 dimensions) showing that for distant observers (in)determinacy is relative. Even if each of their local TRNG outputs a bit, becoming determinate from indeterminate, it is only in the overlap of their future light-cones that both bits become determinate. Note that both a=0 and b=0 are indeterminate in the entire white region.

In our simple example, this means that at 1:00 p.m., the proposition “a=0” has no definite truth value for Bob, nor for Charlie, but it is for them still indeterminate, as it was for Alice before 1:00 p.m. It is only one minute later, i.e., at 1:01 p.m., that “a=0” acquires a definite truth value for Bob, though Bob may still not know this value. For instance, if Bob also holds a TRNG—here assumed to generate randomness locally and independently of Alice’s TRNG—which also outputs a fresh random bit, denoted by *b*, every minute; then, the value of their sum modulo 2, a⊕b, is indeterminate everywhere until 30 s after 1:00 p.m. (in their common inertial frame), and the proposition “a=b” has no truth value, i.e., is indeterminate, until then (see Figure 2).

The present rebuttal of Rietdijk’s and Putnam’s argument is in agreement with that put forward by Stein [26], who also argued for the notion of indeterminacy relative to a space-time point and pointed out the inadequacy of what we have named *present reality*. However, his argument differed from ours inasmuch as it was based on some anthropic considerations on the distance that light can travel within an interval of time that we can perceive as present. Moreover, our view of relative determinacy is also similar to that of Savitt, who—without directly addressing Rietdijk’s and Putnam’s argument—put forward the idea that “special relativistic transience is the successive occurrence of local *nows* along a timelike curve.” [27]. Further, Myrwold [28] discussed the compatibility between relativity and the openness of future (indeterminacy) in the context of objective collapse models of quantum mechanics. Finally, Healey [29] reached a similar conclusion to our relativity of (in)determinacy. However, he used a pragmatist view (which, in contrast to our position, takes probabilities to be relative to hypothetical agents in a specific physical situation, rather than objective propensities) to claim that the assignment of quantum states (and thus, the probabilistic predictions they entail) should be specified relative to different hypothetical observers located at distant space-time regions, in a relativistic scenario.

### 3.2. “Classical” Correlated Randomness

In the previous section, we argued that contrarily to a well-known philosophical argument, indeterminism and relativity remain compatible, insofar as the (in)determinacy of the values taken by physical variables, each measured by a distinct relativistic observer, is relative. So far, however, we have limited our analysis to the assumption that each TRNG generates random bits at its local position and independently from any other random generators. Yet, one can, in principle, envision a form of “classical” correlated randomness between two or more TRNG’s, which will help bridge the gap between the classical and the quantum case. (Here, by “classical” we mean without introducing the structure of quantum probabilities, but merely consider systems whose outcomes are correlated through randomness).

Let us thus consider once more two distant observers, Alice and Bob, each provided with a TRNG such that their respective bits *a* and *b* take one of their two possible values with equal probability, i.e., $p\left(a=0\right)=p\left(a=1\right)=\frac{1}{2}=p\left(b=0\right)=p\left(b=1\right)$. However, when considered jointly, some correlations between the bits outputted by the two TRNGs may have been established. (We do not discuss possible physical mechanisms that may establish these kinds of correlations, but we assume that in principle these can exist). For example, although at the local level the values of the bits are random with uniform distribution, there could be a bias towards certain joint results. For instance, the cases when their values are the same could occur with a higher probability, e.g., $p\left(a=b\right)=\frac{3}{4}$. One can think that at the ontological level there is a physical property such as a (nonlocal) propensity—i.e., an objective tendency that quantifies the bias towards the possible realization of an indeterministic outcome—that accounts for the correlations between the two TRNGs. (This is inspired by the propensity interpretation of probability, which was introduced by Popper [30]; see also [5] for a recent application of this concept to indeterministic physics). Due to the spacelike separation between Alice and Bob, there exist inertial reference frames in which Alice’s TRNG generates a random bit, say a=0, before Bob’s one (which thus remains indeterminate in such reference frames). However, because of the correlations between TRNG’s, within the future light-cone of Alice, the propensity for Bob to find the outcome 0 is updated from $p\left(b=0\right)=\frac{1}{2}$ to $p\left(b=0|a=0\right)=\frac{3}{4}$. On the other hand, in a reference frame in which the realization of a fresh bit by Bob’s TRNG comes first, it is the propensity for the outcome of Alice’s TRNG that is updated according to the established correlations. Note that the realization of a fresh bit by a local TRNG, say, on Alice’s side, does not directly affect the value of the bits generated on Bob’s side (otherwise, this could be used to signal). It is Alice’s propensity for the outcome of Bob’s TRNG that is updated, such that if she obtains a=0, within her future light-cone, the propensity associated with the statement b=0 will change (to 3/4 in this case). In this way, when Alice’s bit becomes determinate, the propensity for the value of *b* relative to Alice’s future light cone also becomes determinate, but not the value of *b*. It is only within the intersection of the future light-cones of Alice and Bob that their outcomes unambiguously assume a definite value, which ought to comply with this nonlocal propensity.

The reader acquainted with quantum physics would have already noticed the similarity of this “classical” nonlocal randomness with quantum correlations. However, we wanted here to express a hypothetical property of indeterminacy that can be conceptually introduced independently of quantum theory (although we do observe it experimentally in quantum systems and not in classical ones). Before moving to the quantum case, we deem it useful to consider the so-called Popescu–Rohrlich (PR) boxes [31], because, contrary to the indeterministic scenarios so far discussed (in terms of TRNG), they admit the choice of inputs that is crucial in the quantum case (i.e., the choice of the measurement basis). PR boxes are a theoretical model of an operational setup that displays the maximal amount of correlated (nonlocal) randomness, which, however, does not lead to instantaneous signaling (i.e., it respects the no-signaling conditions). In terms of correlations between two distant parties—which carry out local operations (i.e., they choose an input bit *x* and *y*, respectively) and measure an outcome of some not fully specified random process (indicated by the bits *a* and *b*, respectively)—the no-signaling conditions are given by the fact that the input choice (which, in turn, could be picked according to local and independent TRNGs) of one party cannot directly influence the outcome of the other one. PR boxes are a set of correlations p(a,b|x,y) defined as follows: if the pair of inputs, (x,y)∈{(0,0),(0,1),(1,0)}; then, the outputs are perfectly correlated, i.e., p(0,0|x,y)=p(1,1|x,y)=1/2. Otherwise, if the input pair (x,y)=(1,1), the outcomes are perfectly anticorrelated, i.e., p(0,1|1,1)=p(1,0|1,1)=1/2. It is straightforward to show that these correlations respect the no-signaling conditions. Accordingly, if, for example, x=0 and a=0, then the propensity $p^{A}\left(b|x,y,a\right)=1$ (i.e., in this case, b=0), where the superscript index indicates Alice’s future light-cone, but p(b|y)=1/2 everywhere else.

### 3.3. Quantum Correlated Randomness

In the previous sections, we have discussed different “classical” scenarios that bring together true randomness (i.e., indeterminism) and special relativity, reaching the conclusion that (in)determinacy is relative. Moreover, in the case of correlated randomness between two TRNGs, there are regions of space-time in which different inertial observers attribute, in general, different probabilities for measurement outcomes (corresponding to different objective propensities), and it is only in the overlap between their future light-cones that their predictions match (where they also become testable). However, it would be impossible to discuss indeterminism without addressing quantum physics, which not only is the most successful theory ever in terms of predictions, but also a theory that hints at the indeterministic nature of our world. Indeed, the violation of Bell’s inequalities [32] has proven that if there is at least a random event in the universe, then there can be arbitrarily many of them [33] (see also [5] for a discussion), and even that classical trajectories of particles cannot exist predetermined (if one upholds locality) [34]. Moreover, in a recent work, Dragan and Ekert showed that elementary special relativistic considerations can lead to quantum randomness [35].

Hence, we now introduce quantum (nonlocal) correlations—that is, what happens if Alice’s and Bob’s TRNGs are entangled? Assume an initial maximally entangled state, say, the singlet $|\Psi^{-}\rangle =1/\sqrt{2}\left(|0\rangle |1\rangle -|1\rangle |0\rangle \right)$, where |0〉 and |1〉 are eigenstates of $\sigma_{z}$, the first qbit is with Alice, and the second with Bob. When Alice measures her local qbit on an arbitrary basis, say, the *x*-basis (recall that $|\Psi^{-}\rangle$ has the same form in every basis), she obtains, for example, the outcome a=0, corresponding to the state ${|0\rangle }_{x}$. While Bob’s local quantum state remains unchanged, Alice describes, after her measurement, the global state using the projection postulate, i.e., it becomes ${|0\rangle }_{x}{|1\rangle }_{x}$. Symmetrically, however, Bob locally measures his qbit, which is still in the initial entangled state $|\Psi^{-}\rangle$, in the *y*-basis, and obtains, say, outcome b=0, corresponding to ${|0\rangle }_{y}$. By using the projection postulate, he updates the global state to ${|1\rangle }_{y}{|0\rangle }_{y}$, while Alice’s state remains unchanged. Since Alice’s and Bob’s measurements are carried out in spacelike separation, there are inertial reference frames in which one measurement occurs before the other and vice versa, from which we conclude that there are certain regions of space-time in which the global quantum state as assigned by Alice and Bob differ. At the intersection of the respective future light-cones, however, the two bits acquire their value and the quantum state is reduced to ${|0\rangle }_{x}{|0\rangle }_{y}$, which reconciles with the usual projection postulate. In the way here described, the state is well-defined at every point in space-time, but in every inertial frame there are regions of space-time where two different states are attributed to the two qbits. Notice that the probabilities of the two outcomes *a* and *b* are correlated, as is usual in quantum mechanics (if one performs local measurement in two bases that are not mutually unbiased): this is nonlocal randomness [36], but the states change only locally.

**Figure 3:**
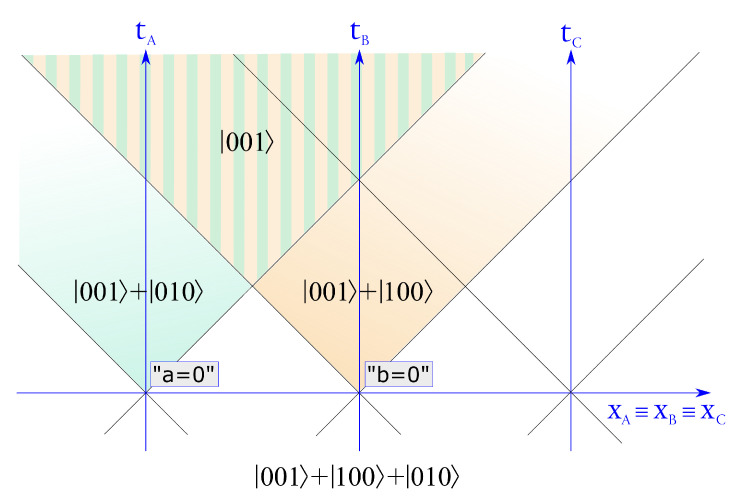
Space-time diagram (in 1 + 1 dimensions) showing a relativistic scenario (readapted from Ref. [38]) in which different global quantum states are assigned in different regions of space-time.

Our view is similar to that of Aharonov and Albert, who, in a series of papers [37,38,39], pointed out the inadequacy of the standard quantum state-vector description for multipartite systems in a relativistic scenario. We thus deem it worth rephrasing, in the language of the present paper, one of the examples given in Ref. [38]. With reference to Figure 3, let Alice, Bob, and Charlie be located at three distant locations and share a tripartite entangled W-state |α〉∝|1〉|0〉|0〉+|0〉|1〉|0〉+|0〉|0〉|1〉, (normalization is omitted) which can be seen as the Fock representation of a single particle in a quantum superposition between the three different locations (entanglement with vacuum). In a certain reference frame, a measurement is performed on the first qbit at $t_{1}$, revealing that the particle is not located at Alice’s position (formally, this means to apply to Alice’s qbit the projector |0〉 〈0|). The global state thus is updated to |β〉∝|0〉(|1〉|0〉+|0〉|1〉). A second measurement is then performed, at time $t_{2}$, on the second qbit, revealing that the particles are also not located at Bob’s location. This leaves the global state in |γ〉=|0〉|0〉|1〉. Given the spacelike separation of these events, however, there exists another reference frame, in which the measurements occur in reversed order. In that frame, the initial state is the Lorentz-transformed of |α〉, which we indicate by $|\alpha^{\prime }\rangle$. The first measurement happens at $t_{2}^{\prime }$ and leads to the updated state $|\eta \rangle \propto |1^{\prime }\rangle \;|0^{\prime }\rangle \;|0^{\prime }\rangle \;+\;|0^{\prime }\rangle \;|0^{\prime }\rangle \;|1^{\prime }\rangle )$ and then, after the second measurement at time $t_{1}^{\prime }$, to the final global state $|\gamma^{\prime }\rangle =|0^{\prime }\rangle \;|0^{\prime }\rangle \;|1^{\prime }\rangle$. This shows that the intermediate states |β〉 and |η〉, which describe the tripartite system in the period in between the two measurements in their respective reference frames, differ in a way that they are not the Lorenz-transformed of one another.

In conclusion, the examples here discussed show that at both the classical level (yet, assuming ontic indeterminacy and associated propensities) and in quantum mechanics, (in)determinacy is in general well-defined only with respect to specific space-time regions. In particular, in this section, we showed that different (global) quantum states pertain to two (or more) spacelike separated regions. It is only at the intersection of the future light-cones of the events that locally determine the truth value of a previously indeterminate empirical statement (i.e., the outcome of a measurement in quantum physics, or the generation of a bit in a TRNG in our previous scenarios) that the state becomes unique and reconciles with the standard quantum-state-update rule.

## 4. The Block-Universe Picture(s)

**Figure 4:**
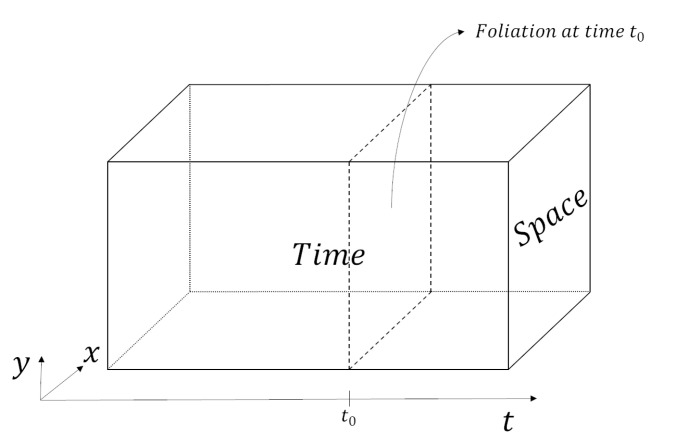
Graphical representation of the “classical” block-universe (in 2 + 1 dimensions).

The proposal expounded in the previous sections shows a possible way to reconcile fundamental indeterminacy and SR. As already recalled, the theory of SR, by putting on the same footing space and time, led to a widely accepted view of a universe that is, at the fundamental level, a “block” in which nothing can really flow or evolve. In this way, the fundamental structure of the universe is regarded as a geometrical (Minkowski) space whose points are events. These events are fixed in this geometrical block and SR only allows them to relate to one another, and the intuitive notion of time is retrieved by fixing a foliation of that space (see Figure 4). We have shown, however, that if one considers the state of (in)determinacy itself as a relative property, then it is not necessary to adopt the block-universe picture.

In more recent years, in the attempt to bring quantum physics into the relativistic world-view, modifications of the block-universe picture have been proposed. In particular, D. Page, W. Wootters, and others [14,40], put forward the view that time in quantum theory—which is usually regarded as a classical scalar parameter entering Schrödinger’s equation—is described by the correlations between a clock (i.e., a physical quantum system) and the (quantum) system whose evolution the clock tracks. In this way, the fundamental object of physics is taken to be a timeless “history state” |Ψ〉〉, which annihilates the total Hamiltonian, i.e.,

(1) $$H|\Psi \rangle \rangle =\left(H_{C}+H_{S}\right)|\Psi \rangle \rangle =0,$$

where $H_{C}$ and $H_{S}$ are the Hamiltonians of the clock and the system, respectively. The standard quantum state is obtained by conditioning the timeless state on the time read by the clock: $|\psi_{S}\left(t\right)\rangle =(|t\rangle ⊗{𝟙}_{S}\Psi \rangle \rangle$. Here, |t〉 is an eigenstate of the operator *T*, such that $[T,H_{C}]=i\hbar$. The unitary evolution of quantum mechanics is retrieved by conditioning the timeless state on two different clock eigenstates (which is, however, not unambiguous, see [41]).

**Figure 5:**
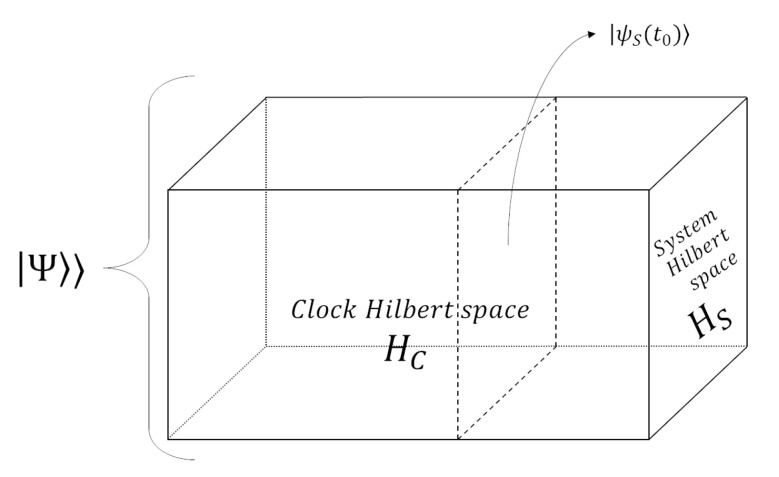
Graphical representation of the “quantum” block-universe as envisioned by the Page–Wootters formalism and the many-worlds interpretation of quantum physics.

Assuming the universality of quantum theory (i.e., that there is, in principle, no limit to its domain of validity), this leads to regarding the timeless “history state of the universe” as *the* fundamental entity of nature. This creates a novel “quantum” block-universe picture, in which what really exists is a single huge quantum “history” state, which encapsulates every event in the universe. The standard notion of time emerges from correlations between clocks and systems (ultimately, all the components of the universe), in a similar fashion to the foliation of the classical block-universe (see Figure 5).

Note that at the interpretational level, the Page–Wootters formalism fits Everett’s relative state interpretation, popularized as the Many-World interpretation of quantum theory [15]. It ought to be remarked that our proposed solution for quantum scenarios (as discussed in Section 3.3) is formally similar to the Many-World interpretation, as both assume the coexistence of different quantum states for the same considered system. However, there is an irreconcilable fundamental difference in our views: we take as a tenet that the outcome of a measurement always yields a single outcome (out of its possible ones), whereas in the Many-World interpretation there is a timeless state of the universe that is branched, bringing into existence at the same time all the possible outcomes at once in parallel “worlds” or realities (Note that, in parlance, the realities are said to be parallel to indicate that the alternative realities coexist; at the formal level, the different branches of the wave function associated with these realities are *orthogonal* in the Hilbert space). The metaphysical consequence of these two views are, thus, not only different but actually antithetical. While in the quantum block-universe picture the main object is the single universal wave function and time plays no fundamental role, in our view, measurement outcomes (e.g., the actualized bits of a TRNG) are genuine acts of creation that transform potentiality into actuality. To this extent, our view departs from any received block-universe picture(s), taking (“creative”) time as a primitive notion.

As such, our relative indeterminacy and the block-universe picture(s), should be considered two alternative world-views that stem from interpreting the theory of special relativity. This resembles the alternative interpretations of the quantum formalism, or the alternative interpretations—deterministic or otherwise—of classical physics as we proposed in Ref. [5].

## 5. Outlook

We have argued that, under the reasonable assumption of finiteness of information density, physics should comply with both indeterminism and relativity. Notwithstanding some historical criticisms, we have shown that these two views are compatible if one regards (in)determinacy itself as relative. Our analysis was based on the formulation of indeterminacy in terms of a third logical truth value (besides “true” and “false”) for empirical propositions. Thus, this is reminiscent of mathematical intuitionism, where the law of the excluded middle fails [11,25].

Despite these promising preliminary conclusions, a number of fundamental questions concerning a hypothetical physical framework that would bring together special relativity and indeterminism (possibly independently of quantum mechanics) remain open. For example, if we enforce the principle of finiteness of information density, the concept of a relativistic “event”, usually defined as a (mathematical) point in the Minkowski space-time, would need to be substituted by a finite hypervolume. This implies that also light-cones would not be perfectly determined, but their edges would be somehow blurred; does this mean that the future determinacy of physical variables is also not perfectly determined? To this end, following the principle of finiteness of information density, we have introduced in previous works [4,5] a concrete model of indeterministic classical (nonrelativistic) physics. This was achieved by replacing the usual real numbers—which are customarily assumed to be the values taken by physical quantities—with what we named “finite information quantities” (FIQs). It would be interesting to analyze in detail the potential consequences of introducing FIQs in the context of special relativity.

Furthermore, in an indeterministic worldview, one would have to reconcile the apparent need for a discretized time—due to a series of genuine acts of creation—with the continuous geometry of space-time entailed by relativity (see [42] for a work in this direction). Note, however, that we do not argue for a fundamental discretization of space-time; we agree that continuity is an irreplaceable feature in relativity theory, but we maintain that the continuum is retrieved similarly to Brouwer’s “viscous continuum” in intuitionistic mathematics, wherein numbers that constitute the continuum are processes that become more and more determinate as time passes [11]. Can this proposed approach in terms of finite information help go beyond the standard relativistic block-universe picture, in which time is a mere illusion, and support instead Reichenbach’s view according to which “the parallelism (between space and time) does not exist objectively and that in natural science, time is more fundamental than space”? [18,43].

## Figures and Tables

**Table 1:** Differences between epistemic (un)certainty and ontic (in)determinacy. The value of a variable *a* can be known only if it is determinate.

	Epistemic	Known *a*	Unknown *a*
Ontic			
Determinate *a*		✓	✓
Indeterminate *a*		✗	✓

## Data Availability

Not Applicable.
